# From Local to International Approach: Prognostic Factors and Treatment Outcomes in Neuroblastoma—A 30-Year Single-Center Retrospective Analysis

**DOI:** 10.3390/children12040525

**Published:** 2025-04-19

**Authors:** Joanna Stankiewicz, Monika Pogorzała, Piotr Księżniakiewicz, Jan Styczyński

**Affiliations:** Department of Pediatric Hematology and Oncology, Collegium Medicum, Nicolaus Copernicus University Torun, Jurasz University Hospital, Sklodowskiej-Curie 9, 85-094 Bydgoszcz, Poland

**Keywords:** neuroblastoma, chemotherapy, therapeutic era, survival

## Abstract

**Background/Objectives**: Over the past three decades, significant progress has been made in understanding the biology of neuroblastoma. The integration of prognostic factors has facilitated risk stratification and the development of targeted treatment approaches. This study aims to analyze the outcomes of pediatric patients with neuroblastoma treated at a single oncology center over a 30-year period. **Methods**: This retrospective study analyzed data from patients aged 0–18 years with neuroblastoma, treated at the Department of Pediatric Hematology and Oncology in Bydgoszcz, Poland, between 1993 and 2023. The study endpoints included the 5-year probability of overall survival (pOS), event-free survival (pEFS), and relapse-free survival (pRFS), analyzed separately for low/intermediate- and high-risk groups. **Results**: Seventy-five patients met the inclusion criteria. Thirty-two children were categorized as high-risk patients and forty-three as low/intermediate risk. During the study period, outcomes in the low/intermediate-risk group improved significantly (the 5-year pOS 85.7% vs. 100.0%, *p* = 0.019; the 5-year pRFS 85.7% vs. 100.0%, *p* = 0.662; the 5-year pEFS 83.3% vs. 100.0%, *p* = 0.038). In the high-risk group, outcomes improved but did not reach statistical significance (the 5-year pOS 0.0% vs. 41.1%, *p* = 0.342; the 5-year pRFS 0.0% vs. 32.5%, *p* = 0.180; and the 5-year pEFS 0.0% vs. 21.5%, *p* = 0.537). Sixteen patients experienced relapse, of whom only three survived; stem cell transplantation at relapse significantly improved survival (OS 0.0% vs. 50.0%, *p* = 0.001). In the multivariable analysis, stage at diagnosis was a prognostic factor for pOS (HR 6.0; 95%CI 0.7–49.6, *p* = 0.096), while pelvic localization was a risk factor for pRFS (HR 3.0; 95%CI 0.8–10.5; *p* = 0.084). **Conclusions**: This analysis highlights significant advancements in the diagnosis and treatment of neuroblastoma. Nevertheless, outcomes for high-risk patients and those who experience relapse remain poor, underscoring the need for further therapeutic improvements.

## 1. Introduction

Neuroblastoma (NBL) is the most common extracranial solid tumor of childhood, characterized by heterogeneous biology and a variable clinical course. The primary tumor arises from primordial neural crest cells and can develop anywhere where sympathetic tissue is present. However, the majority of cases are diagnosed in the adrenal glands or paravertebral ganglia [1,2]. Clinical presentation ranges from an incidentally detected adrenal mass to advanced metastatic disease with systemic manifestations. Moreover, the natural history of neuroblastoma is highly heterogeneous, with some cases exhibiting spontaneous regression or differentiation, while others follow a highly aggressive course with rapid tumor progression and widespread metastasis [1,2,3].

For more than three decades, neuroblastoma treatment has served as a paradigm for tailored, personalized therapy in pediatric oncology [1,4,5,6,7,8,9,10]. Using known biological and clinical factors, patients with neuroblastoma can be divided into three therapeutic groups: low-risk, intermediate-risk, and high-risk. As knowledge about prognostic factors and therapy responses continues to expand, each risk group is further subdivided into smaller, more specific therapeutic subgroups. Although the overall survival rate in the low- and intermediate-risk groups exceeds 90%, high-risk patients have a long-term survival rate of less than 50% [2,5,11,12,13,14]. The implementation of multimodal, intensive chemotherapy, followed by megatherapy with autologous hematopoietic stem cell transplantation (auto-SCT) and maintenance therapy with 13-cis-retinoic acid (13-cisRA), as well as the introduction of immunotherapy with anti-GD2 monoclonal antibodies, has significantly improved outcomes in the HR group [1,6,9,10,11,14,15]. However, children with refractory or relapsed disease have a particularly unfavorable prognosis, and currently, no established effective therapy exists for these patients [16,17,18,19,20]. The gap in our understanding of effective therapies for refractory and relapsed disease remains an important clinical problem.

This study aims to analyze the risk factors, treatment strategies, and outcomes of the entire cohort of pediatric patients with NBL treated at a single oncology center in Poland over a 30-year period. The analysis reflects the expanding knowledge about neuroblastoma biology and the improved therapy results related to the introduction of novel therapeutic modalities in NBL treatment.

## 2. Materials and Methods

This retrospective study analyzed the prognostic factors and outcomes in pediatric patients diagnosed with neuroblastoma and treated in a single tertiary oncology center in Poland during the respective therapeutic periods.

Data from patients aged 0–18 years treated at the Department of Pediatric Hematology and Oncology, University Hospital No. 1 in Bydgoszcz between January 1993 and December 2023 were analyzed. The observations were completed in November 2024. The study included newly diagnosed tumors of neuronal origin that were confirmed as neuroblastomas by pathologists with experience in pediatric malignancies. Clinical data for patients treated between 1994 and 2003 were obtained from traditional medical records, whereas data from 2004 onward were stored and reviewed electronically. Patients were excluded if they had incomplete medical data, were diagnosed with malignancies other than neuroblastoma (e.g., ganglioneuroma, paraganglioma), were lost in follow-up, or were treated at other centers and referred to the Department solely for stem cell transplantation (SCT) as part of therapy.

Patients’ medical histories were reviewed with a particular focus on the diagnosis of cancer-predisposition syndromes before NBL incidence and family history of malignancies. The symptoms presenting at diagnosis included hypertension (blood pressure above the 95 percentile for age and height), tachycardia, pain, constipation, diarrhea, Horner syndrome (unilateral ptosis, anhidrosis, and miosis), signs of spinal cord compression (muscle weakness, sensory deficits, urinary and rectal sphincter dysfunction), and opsoclonus–myoclonus–ataxia (OMA) syndrome.

The histological diagnosis of neuroblastoma was based on conventional tissue-staining histology with additional immunohistochemistry, if indicated. The following NBL subtypes were diagnosed in the analyzed cohort: differentiating NBL, poorly differentiated NBL, undifferentiated NBL, and ganglioneuroblastoma. If the histologic subtype was not indicated in the medical records, neuroblastoma not otherwise specified (NBL NOS) was diagnosed.

At diagnosis, blood tests for hematologic and biochemistry (complete blood count, ferritin, and lactate dehydrogenase levels) and urinary dopamine and catecholamine metabolites, including homovanillic acid (HVA) and vanillylmandelic acid (VMA), were performed. The radiologic work-up included chest X-rays, abdominal ultrasonography (USG), computed tomography (CT), or magnetic resonance imaging (MRI) of the primary tumor with tumor volume calculation and iodine-123-labeled metaiodobenzylguanidine (MIBG) scan (technetium bone scan if primary tumor MIBG negative). Bone marrow involvement was evaluated using bone marrow aspirations and bone marrow trephines (both from two separate evaluable sites). Since 2001, tumor studies have been conducted for the MYC gene copy number status, and since 2015, for genomic copy number profiles with high-resolution array comparative genomic hybridization (aCGH) and ALK gene mutations.

All patients were staged according to the revised International Neuroblastoma Staging System (INNS) [21]. Since 2009, an additional staging system according to the International Neuroblastoma Risk Group Staging System (INRGSS) has been used [22].

The definition of risk groups varies between different protocols and therapeutic periods, as successive research has introduced new prognostic factors for risk group stratifications [5,6,7,8,12,13,14,21,22,23]. Currently, risk group stratification is based on various features related to symptoms, diagnosis, and tumor biology, including tumor genetic profiles. However, many of these factors, measurements, and results were not available during earlier analysis periods, particularly before the 2000s. To enable reliable comparisons between specific therapeutic approaches in this study, patients were divided into two risk groups based on age and disease stage at diagnosis. Patients were classified as low/intermediate risk if they were diagnosed with stage 1–3 neuroblastoma according to INNSS or with stage 4 disease within the first 12 months of life (including stage 4S). Patients older than 12 months with stage 4 disease were classified as high risk.

Patients were treated according to therapeutic protocols dedicated to the relevant risk groups.

The high-risk group was treated according to the Study Group of Japan for Treatment of Advanced Neuroblastoma Tokyo (TOKYO) protocol from January 1993 to January 2002 and the High Risk Neuroblastoma Study 1 (HR-NBL1) of SIOPEN from February 2002 onwards [6,7,9,14,15,24,25].

Patients from the low/intermediate-risk group were treated according to the French Society of Pediatric Oncology Neuroblastoma 90 and 94 therapeutic protocols (SFOP NBL) from January 1993 to October 2002, and a subgroup of patients at stage 1–2 was treated according to the SFOP NBL study to June 2011 [4,5,11,23]. From November 2002 to June 2011, stage 3 patients and patients at stage 4 younger than 12 months were treated according to a Multicenter Study for Infants designed by the International Society of Pediatric Oncology European Neuroblastoma Study Group (SIOPEN INES) [12,13]. From July 2011 onwards, all patients from the low/intermediate-risk group were treated according to the SIOPEN European Low and Intermediate Risk Neuroblastoma study Version 3.0 (LINES 3.0) [8]. One patient from the low/intermediate-risk group was treated according to the TOKYO protocol, and one according to HR-NBL1, based on the individual decision of the therapeutic team.

Surgery of primary tumors was performed according to the respective therapeutic protocols and guidelines. The main recommendations are summarized in Table 1.

Complete resection was defined as the macroscopically complete removal of the tumor, permitting the presence of microscopic residuals. Complete remission (CR) was defined as the absence of tumor in any site. Progression was defined as the appearance of a new site of disease, an increase of any measurable lesion by >25%, or a previous negative marrow positive for tumor during first-line treatment [21]. Relapse was defined as the reappearance of the tumor after achieving CR. Event was defined as relapse, progression, secondary malignancy, or death from any cause. Overall survival (OS) was the time calculated from diagnosis to death or last observation, event-free survival (EFS) included the time from diagnosis to an event, and relapse-free survival (RFS) was calculated as the time from diagnosis to relapse.

The endpoints of the study were a 5-year probability of overall survival (pOS), a 5-year probability of event-free survival (pEFS), and a 5-year probability of relapse-free survival (pRFS).

Survival curves of pOS, pEFS, and pRFS were analyzed according to the Kaplan–Meier method and compared by the log-rank test. All features measured at diagnosis were included in the univariate analysis to determine their impact on outcomes. Factors significant in the univariate analysis were used in the multivariate Cox proportional hazards regression model. A value of *p* < 0.05 was considered statistically significant. Statistical analysis was performed using MedCalc^®^ statistical software Version 23.1.3 (MedCalc Software, Mariakerke, Belgium).

## 3. Results

### 3.1. Patient Characteristics

During the period from January 1993 to December 2023, a total of 113 patients aged 0–18 years were hospitalized in the Department of Pediatric Hematology and Oncology with the diagnosis of tumors of neuronal origin. Among them, 107 were finally diagnosed with neuroblastoma, 4 with ganglioneuroma, and 2 with paraganglioma. Within the 107 neuroblastoma patients, 20 have been treated in different oncology centers and referred to our department for stem cell rescue procedures. Another one was hospitalized solely for the stem cell harvesting procedure without SCT. Eight patients were excluded because of insufficient or incomplete data available, and three patients were lost in follow-up (they were transferred to other oncology centers by their parents’ decision). In total, 75 patients were included in the analysis.

Median age at diagnosis was 1.8 years (range 0.0–12.4 years), and 28 patients (37.3%) were younger than 12 months at diagnosis. None of the analyzed children had a family history of neuroblastoma or other malignancies, and one was earlier diagnosed with cancer predisposition syndrome (neurofibromatosis type 1). However, two patients (a 6-month-old boy and a 4.6-year-old girl) had café au lait spots in clinical examinations at diagnosis. Stage 1 disease was diagnosed in 11 patients (14.7%), stage 2 in 6 (8.0%), and stage 3 in 15 cases (20.0%). Most patients presented with metastatic disease (n = 43, 57.3%), of whom six presented with stage 4S. Thirty-two children were categorized as high-risk patients and forty-three as low/intermediate risk. Patients’ detailed characteristics are shown in Table 2.

### 3.2. Treatment in the Low/Intermediate-Risk Group

In the low/intermediate-risk group, most patients were treated according to the LINES 3.0 therapeutic protocol (n = 20, 46.2%). Complete resection during either primary or delayed surgery was achieved in 28 patients (65.1%). Among these, 18 patients underwent complete macroscopic removal of the primary tumor during initial surgery. Primary surgery served as the definitive therapy in 15 cases, including 12 stage 1 patients, 2 stage 2 patients, and 1 stage 4S patient. Three stage 4S patients had only a biopsy of the primary tumor without further treatment. Radiotherapy was administered as a complementary therapy in eight cases (18.6%).

### 3.3. Treatment in the High-Risk Group

The majority of patients in the high-risk group were treated according to the HR-NBL1 therapeutic protocol (n = 25, 78.1%). All patients were diagnosed based on a biopsy of either the primary tumor or a metastatic site and received primary chemotherapy. Delayed complete resection was achieved in three cases (9.4%). Twenty patients underwent autologous-stem cell transplantation (auto-SCT). In 15 cases, local radiotherapy (RTX) was performed following induction chemotherapy, surgery, and auto-SCT. Sixteen children received maintenance treatment with 13-cis-retinoic acid (13-cisRA), and six patients were eligible to receive dinutuximab beta (anti-GD2 monoclonal antibodies) during maintenance therapy.

### 3.4. Outcomes

Complete remission was achieved by 82.7% of patients (n = 62). For the entire cohort, the 5-year pOS was 66.7%. Patients in the low/intermediate-risk group had a significantly better 5-year pOS compared to those in the high-risk group (91.9% vs. 35.9%, *p* < 0.001). Relapse occurred in 16 cases, with a median time to relapse of 0.8 years (range 0.0–3.2 years). In seven cases, relapse was observed at the primary tumor site, while in five cases, the patients presented with disseminated disease at the time of relapse diagnosis. Characteristics of patients with relapsed disease and details of subsequent therapies are presented in Table 3. Among patients who experienced relapse, only three survived. Stem cell transplantation (SCT) in relapse was the only therapeutic modality that significantly improved outcomes in this group (OS 0.0% vs. 50.0% for patients who underwent SCT in relapse, *p* = 0.001).

The 5-year pRFS for the whole cohort was 68.3%. Patients in the high-risk group had a significantly higher risk of relapse compared to those in the low/intermediate-risk group (the 5-year pRFS 28.3% vs. 94.9%, *p* < 0.001).

The 5-year pEFS for all analyzed patients was 57.3%, with a 5-year pEFS of 94.9% for the low/intermediate-risk group and 28.3% for the high-risk group (*p* < 0.001). Disease progression was observed in five cases. Four patients with progression were treated with chemotherapy, of whom two underwent SCT; one child received palliative care only. None of the patients with disease progression survived. No cases of secondary malignancies were observed.

**Figure 1:**
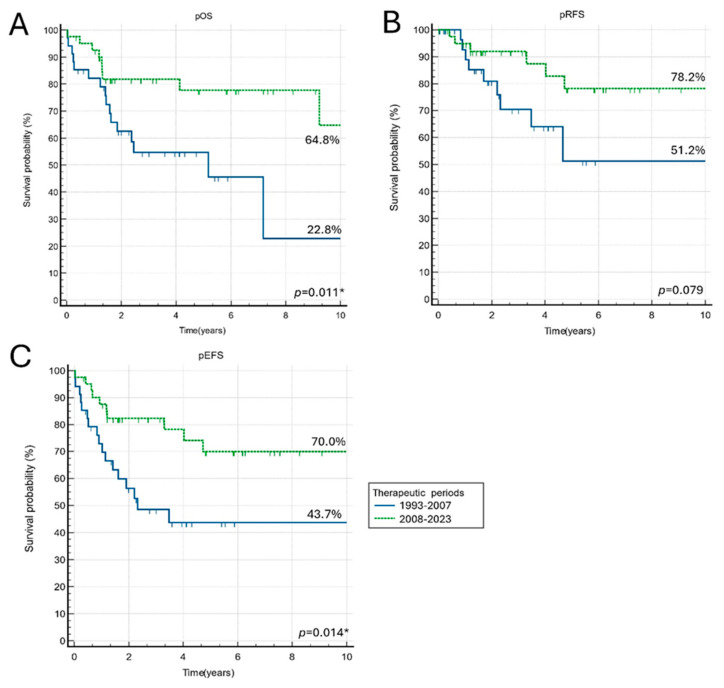
The long-term probability of overall survival (pOS), event-free survival (pEFS), and relapse-free survival (pRFS) divided by therapeutic periods. (**A**) The 10-year pOS for the whole cohort divided by therapeutic periods; (**B**) The 10-year pRFS for the whole cohort divided by therapeutic periods; (**C**) The 10-year pEFS for the whole cohort divided by therapeutic periods. * *p* < 0.05.

Patients treated according to modern therapeutic protocols, utilizing genetic-based stratification, had better outcomes. Patients treated during the second half of the analyzed period have significantly better pOS, pEFS, and pRFS compared to the cohort treated before the year 2008 (Figure 1).

Twenty-four patients died, with the most common causes of death being disease relapse (n = 9, 37.5%) and treatment-related complications (n = 9, 37.5%). Four patients died due to primary disease progression, and in two cases, the cause of death was unknown (both patients died outside our hospital). Among the treatment-related deaths, four were attributed to infectious complications, four to treatment toxicity, and one to complications following primary tumor resection. The median time from diagnosis to death was 1.3 years.

### 3.5. Therapy Results in the Low/Intermediate Group

**Figure 2:**
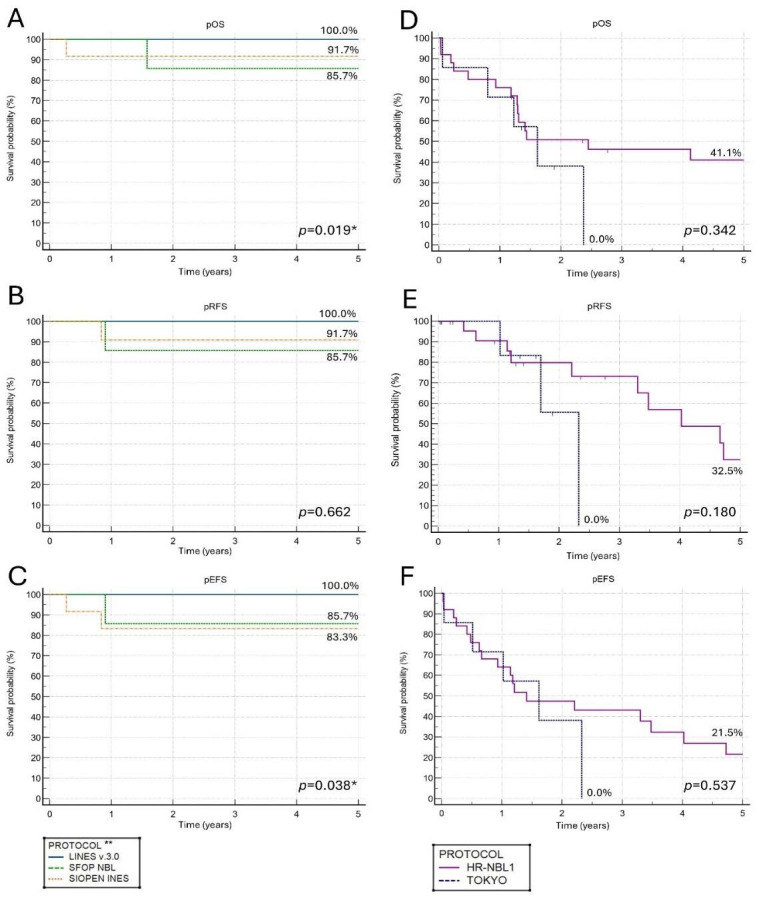
The 5-year probability of overall survival (pOS), event-free survival (pEFS), and relapse-free survival (pRFS) for consecutive therapeutic protocols divided by risk groups. (**A**) The 5-year pOS for low/intermediate-risk group patients according to therapy protocols; (**B**) The 5-year pRFS for low/intermediate-risk group patients according to therapy protocols; (**C**) The 5-year pEFS for low/intermediate-risk group patients according to therapy protocols; (**D**) The 5-year pOS for high-risk group patients according to therapy protocols; (**E**) The 5-year pRFS for high-risk group patients according to therapy protocols; (**F**) The 5-year pEFS for high-risk group patients according to therapy protocols; * *p* < 0.05; ** One patient treated with TOKYO and one patient treated with HR-NBL1 protocol were excluded from the (**A**–**C**).

The differences between therapeutic protocols in 5-year pOS and pEFS were statistically significant, with the best results observed in patients treated according to LINES 3.0 (Figure 2). No progression was observed in this group. One patient treated with the TOKYO protocol died due to treatment-related complications; one patient treated with the HR-NBL1 protocol is alive in remission. Relapse occurred in three patients. After relapse, one patient received chemotherapy only, one received chemotherapy followed by auto-SCT and additional RTX, and one received chemotherapy followed by auto-SCT, RTX, and maintenance with 13-cisRA. Three patients died: one in relapse (patient primary treated with SFOP NBL 94 protocol and with TOKYO after relapse), one due to treatment-related toxicity (infection), and in one case, the cause of death was unknown (the patient died in a different hospital after completion of therapy).

### 3.6. Therapy Results in the High-Risk Group

Figure 2 shows that 5-year pOS was superior in patients treated according to the HR-NBL1 protocol. However, the results did not reach statistical significance. Patients treated with auto-SCT and maintenance with 13-cisRA had a better prognosis (the 5-year pOS 50.3% vs. 24.0%, *p* = 0.001). In the subgroup treated in maintenance with an addition of anti-GD2 monoclonal antibodies, the 5-year pOS was better, but the difference did not reach statistical significance (the 5-year pOS 50.0% vs. 38.8%, *p* = 0.449). However, prolonged observations indicated prominent improvement in long-term outcomes among patients treated with anti-GD2 monoclonal antibodies (the 10-year pOS 50.0% vs. 12.1%).

Thirteen patients experienced relapse, with a median time to relapse of 0.9 years. The differences in 5-year pRFS between protocols were visible but not statistically significant (0.0% vs. 32.5%, *p* = 0.180, Figure 2). None of the therapy modalities used in first-line treatment (auto-SCT procedure, maintenance with 13-cisRA, or anti-GD2 monoclonal antibodies) significantly reduced the probability of relapse.

The 5-year pEFS was 0.0% for TOKYO and 21.5% for the HR-NBL1 protocol (Figure 2). Although therapy results with the auto-SCT procedure were better (the 5-year pEFS 26.8% vs. 20.0%, *p* = 0.001), the outcomes remained poor. The addition of maintenance with 13-cisRA significantly improved the 5-year pEFS (34.8% vs. 0.0%, *p* = 0.001 for 13-cisRA). Patients treated with anti-GD2 monoclonal antibodies had a higher 5-year pEFS, but the differences were not statistically significant (50.0% vs. 13.8%, *p* = 0.308).

### 3.7. Prognostic Factors

**Figure 3:**
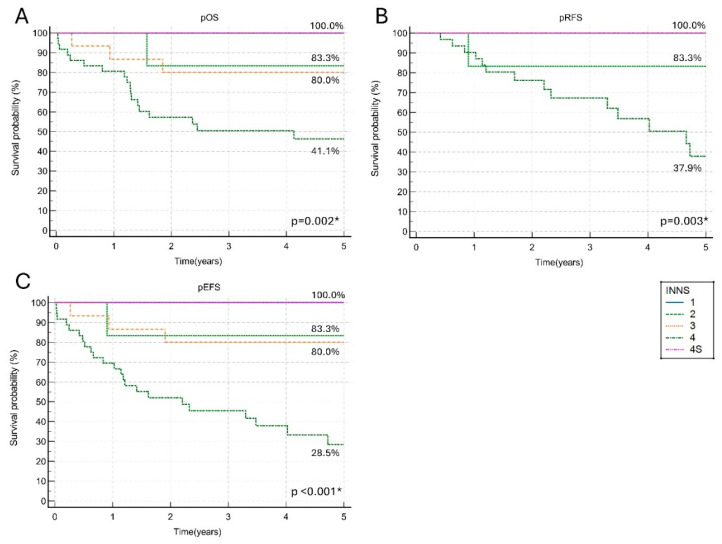
The outcomes according to five consecutive International Neuroblastoma Staging System (INNS) disease stages. (**A**) The 5-year probability of overall survival (pOS) for five consecutive INNS disease stages; (**B**) The 5-year probability of relapse-free survival (pRFS) for five consecutive INNS disease stages; (**C**) The 5-year probability of event-free survival (pEFS) for five consecutive INNS disease stages. * *p* < 0.05.

Stage at diagnosis was a significant prognostic factor. Patients with stage 1 and 4S disease had an excellent 5-year pOS and pEFS of 100.0%. In patients with stage 2 and 3 neuroblastoma, the 5-year pOS and pEFS reached 80.0% or above, while patients with stage 4 neuroblastoma had the 5-year pOS of 46.3%, the 5-year pRFS of 37.9%, and the 5-year pEFS of 28.5% (Figure 3).

Age above 12 months at diagnosis was a risk factor for death (OR 7.9, 95% CI 2.1 to 29.9, *p* = 0.002), event (OR 6.8, 95% CI 2.1 to 22.7, *p* = 0.014), and relapse (OR 13.6, 95% CI 1.7 to 109.3, *p* = 0.014). Other significant risk factors included symptoms such as pain, hypertension, and tachycardia at diagnosis, increased LDH and ferritin levels, poorly differentiated histology, primary tumor localization in the pelvis or retroperitoneum, MIBG-positive tumors, N-MYC gene amplification, and structural chromosomal alterations (SCA) in tumor tissue (Table 4). In the multivariable analysis, INNS stage was a risk factor of borderline significance for pOS and pEFS, while pelvis localization was a factor of borderline significance for pRFS (Table 5).

## 4. Discussion

This 30-year, single-center analysis reflects significant improvements in the diagnostic and therapeutic approaches for NBL. As Poland has never established a national therapeutic program for neuroblastoma, our department has used therapeutic protocols from other countries [4,5,6,7,8,9,11,12,13,14,15,23,26]. In the 1990s, patients were treated using Japanese and French protocols. The results obtained in our cohort were inferior to those reported by the Neuroblastoma Study Group of the Société Francaise d’Oncologie Pédiatrique and the Study Group of Japan [4,6,14,15]. Poorer outcomes could be attributed to limited access to detailed therapy guidelines and high rates of treatment-related mortality. Since the early 2000s, patients have been treated according to international therapy protocols, leading to significant improvements in treatment outcomes.

During the study period, diagnostic and therapeutic recommendations have evolved into more specific and comprehensive guidelines, enabling precise execution of all therapeutic stages. An excellent example of the development is surgical guidelines. In the 1980s and 1990s, the surgery range and acceptable extension of the surgical site, as well as surgery timing, were assessed individually by surgeons [6]. Currently, the surgical guidelines are detailedly described in therapeutic protocols, with well-recognized image-defined risk factors and surgery time [1,2,7,8,27]. Moreover, the final decision regarding the surgical range is made by an interdisciplinary team comprising a pediatric oncologist, radiologist, and surgeon. Multidisciplinary collaboration plays a crucial role in the preparation of comprehensive treatment plans while minimizing postoperative complications.

In the last three decades, significant progress has been made in our understanding of neuroblastoma biology, particularly regarding the genetic and molecular background of the tumor and its distinct natural course in infants [2,3,12,22]. The stage at diagnosis is one of the most important prognostic factors. Prognostic factors other than age and stage include elevated LDH and ferritin levels, segmental chromosomal aberrations, MYC oncogene amplification, and unfavorable histology [1,2,8,13,16,23]. Although most risk factors were also identified in this study, in our cohort, clinical manifestations, such as hypertension and pain, were associated with decreased pOS, pRFS, and pEFS, whereas tachycardia was specifically linked to lower pEFS in a univariate analysis.

The integration of prognostic factors into clinical practice has facilitated effective risk stratification, allowing for adjusted treatment approaches. In the analyzed cohort, patients in the low/intermediate-risk group had a favorable prognosis, with a 5-year pOS and pEFS above 80.0%. A subgroup of patients treated with the LINES 3.0 protocol achieved excellent outcomes, with a 5-year pOS and pEFS of 100.0%. Significant progress in therapeutic outcomes for the low- and intermediate-risk groups was achieved by minimizing invasive treatment in subgroups with highly favorable prognoses while intensifying therapy for patients with identified risk factors. This risk-adapted strategy has optimized treatment intensity while reducing toxicity and improving overall patient outcomes.

Intensive treatment regimens also resulted in gradual improvements in survival among children in the high-risk group. Although overall outcomes in this group remained unfavorable, the 5-year pOS of the analyzed cohort increased from 0.0% to 41.1% during the study period. The TOKYO protocol was one of the first to incorporate multimodal, intensive chemotherapy for high-risk patients [6,14,15]. Although the results of the protocol were unsatisfactory in our department, it paved the way for further intensive treatment according to the HR-NBL1 protocol, which yielded significantly better outcomes. Therapy with anti-GD2 monoclonal antibodies did not significantly improve pOS and pEFS in our cohort. However, prolonged observation suggests that the addition of anti-GD2 monoclonal antibodies improves long-term outcomes.

More than half of children with high-risk neuroblastoma either fail to respond to standard therapies or experience relapse [16,19,20,28]. Patients with relapse have particularly unfavorable outcomes, with long-term pOS ranging from 14.1% to 20.0% [17,18,19,20]. Although no standard second-line therapy has been established, limited evidence indicates improved survival following myeloablative chemotherapy with auto-SCT [20,29]. This trend was also observed in our study, with SCT in relapse being the only therapeutic modality that significantly improved outcomes. Nevertheless, the choice of bridging therapy remains a topic of discussion, as reflected by the variety of salvage chemotherapy used during the study period. Regimens containing temozolomide and topotecan are currently the standard backbone of bridging chemotherapy [17,18,30]. However, most second-line therapy trials for neuroblastoma have been single-arm studies, lacking a valid comparator and primarily assessing short-term outcomes, such as response rate [17,18,30]. Given the heterogeneity of the disease and second-line therapy, there is limited knowledge of which patients are more likely to respond to treatment and achieve long-term survival after primary therapy failure [16,17,29].

Due to the retrospective nature of the analysis, this study has some obvious limitations. Some patients were excluded from the analysis due to missing medical data. The group comprised heterogeneous patients treated within 30 years, and the number of patients treated according to consecutive protocols was relatively small. Although some genetic diagnostic tests were not available in the early period of the analysis, the most important information about the clinical and laboratory results was included. Moreover, the lack of genetic testing reflects the limited knowledge about neuroblastoma biology in the 1990s and early 2000s. Furthermore, the endpoints were achieved in all analyzed cases, which enabled a comparison between therapeutic approaches. The data from this study may serve as a background for further research, particularly those focusing on relapsed disease.

## 5. Conclusions

This study highlights the significant progress that has been made in the diagnosis and treatment of neuroblastoma. The development of modern diagnostic methods, including genetic testing of tumor tissues, has facilitated precise risk stratification. Tailored treatment approaches, focused on minimizing invasive interventions for subgroups with highly favorable prognoses while intensifying therapy for high-risk patients, have optimized treatment strategies and improved overall patient outcomes. In our department, treatment guidelines have evolved from individualized therapeutic decisions based on scientific reports to standardized protocols within international trials. However, prognosis remains poor for high-risk patients and those who experience relapse, emphasizing the need for further therapeutic improvements. The lack of standardized second-line treatment guidelines for patients with relapse underscores the urgency for additional clinical trials. With the upcoming SIOPEN HR-NBL-2 trial on the horizon, we expect further improvements in outcomes for patients with high-risk neuroblastoma.

## Figures and Tables

**Table 1:** The main recommendations for surgery according to the analyzed therapeutic protocols.

Therapeutic Protocols	Surgery Guidelines
**TOKYO ^1^**	The timing and method of resection (gross complete resection, partial resection, or biopsy) were determined by each individual institution. Surgery of the primary tumor and lymph node metastases was performed between chemotherapy courses [6,14,15].
**HR-NBL1 ^2^**	CME ^3^ of the primary tumor and involved lymph nodes was encouraged, ideally before SCR ^4^. The operation was to be postponed if nephrectomy was necessary. Tumor resection was permitted at one of three defined time points: within 60 days after the end of induction therapy, after 60 days of induction completion, followed by a topotecan, vincristine, and doxorubicin chemotherapy course, or after SCR [7].
**SFOP NBL 90 ^5^ and SFOP NBL 94 ^6^**	The final decision regarding the extent of surgery (primary or delayed excision attempt versus partial resection or biopsy) was made by an interdisciplinary team comprising a pediatric oncologist, radiologist, and surgeon. A localized tumor was considered unresectable if it crossed the midline, infiltrated major vessels, or posed a high risk of major surgical complications or macroscopically incomplete resection. Surgery with the risk of major organ removal (e.g., kidney, bladder, ureter) was not recommended unless initial chemotherapy had been administered before [4,5,23].
**SIOPEN INES ^7^**	Primary surgery could be performed as complete, near-complete, or incomplete excision of the tumor mass, or it may be limited to an open or needle-core biopsy, depending on the objective and subjective SRFs ^8^ defined based on imaging characteristics. Objective SRFs include infiltration of or close relation to major blood vessels, infiltration of intervertebral foramina, or crossing the midline. The protocol also defines SRFs related to the specific localization of the primary tumor. Subjective SRFs include the ratio between tumor and child size as well as tumor fragility. In the presence of SRFs, primary biopsy was encouraged [12,13].
**LINES 3.0 ^9^**	Primary resection was indicated for patients with localized tumors without IDRFs ^10^ as listed in the study (e.g., tumor encasing major arteries and vessels, tumor encasing vital neural structures such as brachial plexus roots, invasion of more than one-third of the spinal canal, tumor encasing the trachea or principal bronchi, and infiltration of adjacent organs and structures). Biopsy at presentation was indicated for localized tumors with IDRFs and metastatic tumors. Excision of the primary tumor may serve as an alternative diagnostic procedure to biopsy in metastatic tumors, provided the primary tumor is IDRF-negative. The decision to perform delayed resection was made individually based on the child’s age and the presence of IDRFs, as defined in the protocol [8].

^1^ TOKYO—The Study Group of Japan for Treatment of Advanced Neuroblastoma Tokyo; ^2^ HR-NBL1—the High Risk Neuroblastoma Study 1 of The International Society of Paediatric Oncology European Neuroblastoma (SIOPEN); ^3^ complete macroscopic excision; ^4^ stem cell rescue; ^5^ SFOP NBL 90—The French Society of Pediatric Oncology Neuroblastoma 90 therapeutic protocol; ^6^ SFOP NBL 94—The French Society of Pediatric Oncology Neuroblastoma 94 therapeutic protocol; ^7^ SIOPEN INES—a Multicenter Study for Infants designed by the International Society of Pediatric Oncology European Neuroblastoma Study Group; ^8^ SRF—surgical risk factor; ^9^ LINES 3.0—The International Society of Paediatric Oncology European Neuroblastoma (SIOPEN) European Low and Intermediate Risk Neuroblastoma study Version 3.0; ^10^ IDRF—image-defined risk factor.

**Table 2:** Patients’ characteristics considering respective therapeutic protocols.

	TOKYO ^11^	HR-NBL1 ^12^	SFOP NBL ^13^	SIOPEN INES ^14^	LINES 3.0 ^15^
Number of patients	8	27	9	12	19
Male	4	14	5	6	14
Female	4	13	4	6	5
Age (years) mean range	5.5 1.0–12.4	3.8 0.4–10.5	2.7 0.0–12.4	1.3 0.1–7.4	1.5 0.0–11.5
WBC ^1^ (×10^3^) mean range	9.21 2.60–17.00	7.49 4.41–12.7	9.21 4.80–18.00	10.73 8.60–12.40	10.18 2.96–15.40
PLT ^2^ (×10^3^) mean range	322 107–653	301 48–536	502 259–780	379 161–647	488 254–849
HGB ^3^ (g/L) mean range	9.9 7.0–13.5	8.9 4.8–12.4	11.7 9.3–13.4	10.7 9.6–13.2	11.3 7.4–15.9
LDH ^4^ (IU/L) mean range	1313 403–4258	1613 168–7029	548 410–740	627 282–1360	344 223–874
Ferritin(ng/mL) mean range	488 271–706	538 25–2480	85 37–181	119 20–292	97 6–298
Histology					
Differentiating NBL ^5^	2	2	3	1	1
Poorly differentiated NBL	0	5	1	6	12
Undifferentiated NBL	1	4	0	0	2
Ganglioneuroblastoma	0	6	2	2	2
NBL NOS ^6^	5	10	3	3	2
Catecholamine metabolites	4	20	3	7	11
MIBG ^7^ positive tumors	5	18	4	9	10
N-MYC amplification	NA ^10^	13	NA	3	1
SCA ^8^	NA	7	NA	NA	5
NCA ^9^	NA	3	NA	NA	7
ALK gene mutation	NA	0	NA	NA	0
Symptoms					
Hypertension	2	6	1	2	1
Tachycardia	2	3	0	0	1
Pain	7	14	1	4	2
Constipation	3	3	1	3	1
Diarrhea	1	0	0	2	3
Horner syndrome	0	0	0	1	0
Spinal cord compression	2	5	0	3	1
Opsoclonus–myoclonus–ataxia	0	0	0	0	0
Primary tumor localization					
Adrenal gland	6	22	8	7	16
Retroperitoneal	6	22	1	7	5
Mediastinal	2	12	1	5	1
Pelvis	2	12	0	4	1
Neck	1	3	0	0	0

^1^ WBC—white blood cells; ^2^ PLT—platelet count; ^3^ HGB—hemoglobin; ^4^ LDH—lactate dehydrogenase; ^5^ NBL—neuroblastoma; ^6^ NOS—not otherwise specified; ^7^ MIBG—iodine-123-labeled metaiodobenzylguanidine; ^8^ SCA—structural chromosomal alterations, ^9^ NCA—numerical chromosomal alterations; ^10^ NA—not applicable; ^11^ TOKYO—The Study Group of Japan for Treatment of Advanced Neuroblastoma Tokyo; ^12^ HR-NBL1—the High Risk Neuroblastoma Study 1 of The International Society of Paediatric Oncology European Neuroblastoma (SIOPEN); ^13^ SFOP NLB—The French Society of Pediatric Oncology Neuroblastoma 90 and 94 therapeutic protocols; ^14^ SIOPEN INES—A Multicenter Study for Infants designed by the International Society of Pediatric Oncology European Neuroblastoma Study Group; ^15^ LINES 3.0—The International Society of Paediatric Oncology European Neuroblastoma (SIOPEN) European Low and Intermediate Risk Neuroblastoma study Version 3.0.

**Table 3:** Detailed characteristics of patients with relapse.

No.	Sex	Age at Diagnosis (yrs)	Therapy Protocol in 1st Line	Time from Start of Treatment to Relapse (yrs)	2nd Line Chemotherapy	RTX ^1^	Auto-SCT ^2^	13-cisRA ^3^	Anti-GD2 ^4^	MIBG ^5^	Outcome	Time from Relapse to Death (yrs)
1.	M	7.6	TOKYO ^6^	0.9	2nd therapy with TOKYO protocol	0	0	0	0	0	Death (disease progression)	0.1
2.	M	12.4	TOKYO	0.2	SPIC ^7^	0	0	0	0	0	Death (disease progression)	0.2
3.	F	0.1	SIOPEN INES ^8^	0.1	HR-NBL1 ^9^	1	1	0	0	0	Alive	NA
4.	M	0.5	HR-NBL1	0.1	Palliative care	0	0	0	0	0	Death (disease progression)	0.0
5.	F	2.1	TOKYO	0.0	Palliative care	0	0	0	0	0	Death (disease progression)	0.2
6.	F	8.3	SFOP NBL ^10^	1.0	TOKYO	1	0	0	0	0	Death (disease progression)	1.7
7.	M	3.2	HR-NBL1	2.3	TVD ^11^, Cisplatin, Etoposide + ZVAC ^12^ + Temozolomide	0	0	0	0	0	Death (disease progression)	1.7
8.	F	4.6	HR-NBL1	0.3	SIOPEN INES	0	0	0	0	0	Death (disease progression)	0.3
9.	M	1.7	HR-NBL1	3.1	CIT ^13^, VOIT ^14^, ICE ^15^ + Topotecan	0	1	0	0	0	Death (disease progression)	2.5
10.	M	0.1	HR-NBL1	2.0	ICE + Topotecan, TOTEM ^16^	0	1 *	1	0 **	1	Death (TRM ^17^—infection)	5.9
11.	M	0.9	SIOPEN INES	1.1	CIT, ICE+ Topotecan	1	1	1	0	0	Alive	NA
12.	F	2.5	HR-NBL1	2.7	Cyclophosphamide + Vincristine	0	0	0	0	0	Death (TRM—treatment toxicity)	0.1
13.	F	3.1	HR-NBL1	3.2	Cyclophosphamide + Topotecan, Temozolomide + Irinotecan	1	1 *	1	1	1	Alive	NA
14.	M	3.1	HR-NBL1	0.6	Bevacizumab + Irinotecan + Temozolomide	0	0	0	0	0	Death (disease progression)	0.7
15.	F	0.1	HR-NBL1	0.1	Etoposide + Carboplatin + Doxorubicin	1	0	0	0	0	Death (disease progression)	0.1
16.	M	0.3	HR-NBL1	0.1	TOTEM	0	0	0	0	0	Death (disease progression)	0.2

^1^ RTX—radiotherapy; ^2^ Auto-SCT—autologous stem cell transplantation; ^3^ 13-cisRA—13-cis-retinoic acid; ^4^ Anti-GD2—anti-GD2 monoclonal antibodies; ^5^ MIBG—I-131-labeled metaiodobenzylguanidine therapy; ^6^ TOKYO—The Study Group of Japan for Treatment of Advanced Neuroblastoma Tokyo; ^7^ SPIC—sequential postoperative intraperitoneal chemotherapy; ^8^ SIOPEN INES—a Multicenter Study for Infants designed by the International Society of Pediatric Oncology European Neuroblastoma Study Group; ^9^ HR-NBL1—the High Risk Neuroblastoma Study 1 of The International Society of Paediatric Oncology European Neuroblastoma (SIOPEN); ^10^ SFOP NBL—The French Society of Pediatric Oncology Neuroblastoma 90 and 94 therapeutic protocols; ^11^ TVD—topotecan, vincristine, doxorubicin; ^12^ ZVAC—dexrazoxane, vincristine, doxorubicin, cyclophosphamide; ^13^ CIT—carboplatin, irinotecan, temozolomide; ^14^ VOIT—vincristine, oral irinotecan, temozolomide; ^15^ ICE—ifosfamide, carboplatin, etoposide; ^16^ TOTEM—temozolomide and topotecan; ^17^ TRM—treatment-related mortality; * patients received two auto-SCTs, ** patient was disqualified from a therapy with anti-GD2 monoclonal antibodies due to restrictive lung disease as a complication of first-line treatment.

**Table 4:** Univariate analysis of risk factors.

	5-Year pOS ^1^	pOS; *p*=	5-Year pEFS ^2^	pEFS; *p*=	5-Year pRFS ^3^	pRFS; *p*=
Male Female	75.0% 57.0%	0.693	62.0% 52.1%	0.893	70.6% 65.1%	0.854
Age < 12 months Age > 12 months	88.2% 54.1%	0.003 *	84.4% 42.4%	0.002 *	96.2% 52.5%	0.003 *
LDH ^4^ high level LDH normal level	54.8% 82.0%	0.019 *	38.5% 82.3%	0.005 *	51.9% 88.1%	0.066
Ferritin high level Ferritin normal level	53.1% 88.7%	0.005 *	38.0% 88.9%	0.001 *	49.7% 96.0%	0.004 *
Histology						
Differentiating NBL ^5^ Histology other than differentiating NBL	64.8% 66.9%	0.955	64.4% 56.3%	0.807	80.0% 66.3%	0.504
Poorly differentiated NBL Histology other than poorly differentiated NBL	87.1% 58.5%	0.042 *	87.1% 45.9%	0.011 *	100.0% 56.4%	0.006 *
Undifferentiated NBL Histology other than undifferentiated NBL	68.2% 57.1%	0.692	57.1% 57.6%	0.813	71.4% 68.1%	0.581
Ganglioneuroblastoma Histology other than ganglioneuroblastoma	63.7% 81.5%	0.897	61.7% 56.5%	0.737	68.6% 68.7%	0.521
Catecholamine metabolites positive Catecholamine metabolites negative	64.8% 84.4%	0.129	49.4% 85.7%	0.057	59.7% 83.6%	0.232
N-MYC ^6^ positive N-MYC negative	55.7% 87.6%	0.047 *	57.9% 71.9%	0.199	65.3% 82.1%	0.090
SCA ^7^ SCA negative	59.3% 100.0%	0.026 *	49.4% 100.0%	0.016 *	70.3% 100.0%	0.125
NCA negative ^8^ NCA positive	42.9% 84.6%	0.351	42.9% 84.6%	0.085	0.0% 92.3%	0.425
Symptoms at diagnosis						
Hypertension present Hypertension absence	32.1% 73.3%	0.002 *	18.8% 63.5%	0.003 *	31.5% 70.0%	0.030 *
Tachycardia present Tachycardia absent	44.1% 68.8%	0.163	22.2% 60.0%	0.148	22.2% 71.1%	0.003 *
Pain present Pain absent	44.2% 81.9%	0.001 *	26.2% 76.8%	<0.001 *	37.1% 81.3%	0.001 *
Constipation present Constipation absent	48.2% 69.1%	0.497	50.0% 56.9%	0.833	60.0% 66.6%	0.533
Diarrhea present Diarrhea absent	66.7% 66.6%	0.979	66.7% 55.4%	0.940	100.0% 63.5%	0.223
Spinal cord compression present Spinal cord compression absent	72.2% 65.2%	0.847	58.3% 55.7%	0.931	72.7% 64.3%	0.924
Primary tumor localization						
Adrenal gland Other than adrenal gland	67.0% 68.5%	0.514	54.2% 69.5%	0.515	66.3% 72.7%	0.909
Retroperitoneal Other than retroperitoneal	53.1% 86.8%	0.007 *	41.2% 81.0%	0.004 *	56.8% 82.6%	0.140
Pelvis Other than pelvis	44.9% 73.2%	0.021 *	29.8% 65.7%	0.003 *	44.5% 74.1%	0.023 *
Neck Other than neck	25.0% 70.6%	0.133	25.0% 60.3%	0.193	33.3% 71.4%	0.182

^1^ pOS—probability of overall survival; ^2^ pEFS—probability of event-free survival; ^3^ pRFS—probability of relapse-free survival; ^4^ LDH—lactate dehydrogenase; ^5^ NBL—neuroblastoma; ^6^ N-MYC gene amplification; ^7^ SCA—structural chromosomal alterations; ^8^ NCA—numerical chromosomal alterations; * *p* < 0.05.

**Table 5:** Multivariable analysis of risk factors.

Independent Variable	pOS ^4^ HR ^5^	pOS HR 95% CI ^7^	pOS *p*=	pEFS ^8^ HR	pEFS HR 95% CI	pEFS *p*=	pRFS ^9^ HR	pRFS HR 95% CI	pRFS *p*=
Hypertension	3.7	0.7 to 19.6	0.124	2.9	0.7 to 12.9	0.142	1.3	0.2 to 7.7	0.741
Pain	1.0	0.2 to 6.3	0.992	1.2	0.2 to 5.4	0.816	1.7	0.5 to 5.8	0.415
Tachycardia	NA ^6^	NA	NA	NA	NA	NA	2.0	0.3 to 11.5	0.447
Ferritin high level	3.1	0.3 to 30.0	0.334	3.5	0.4 to 32.5	0.266	1.4	0.1 to 14.2	0.771
LDH ^1^ high level	0.4	0.5 to 3.2	0.381	0.6	0.1 to 4.5	0.640	NA	NA	NA
N-MYC ^2^ positive	1.2	0.3 to 4.6	0.780	NA	NA	NA	NA	NA	NA
Pelvis	1.9	0.4 to 10.7	0.447	2.0	0.5 to 7.9	0.319	3.0	0.8 to 10.5	0.084 *
Retroperitoneal	1.5	0.2 to 15.3	0.714	0.9	0.1 to 7.9	0.973	NA	NA	NA
INNS ^3^	6.0	0.7 to 49.6	0.096 *	7.4	0.9 to 57.5	0.056 *	2.9	0.7 to 12.5	0.134
Age < 12 months	0.2	0.1 to 3.9	0.301	0.2	0.1 to 3.7	0.306	3.4	0.3 to 34.8	0.670
Poorly differentiated histology	0.4	0.1 to 3.1	0.377	0.2	0.1 to 1.6	0.149	0.8	0.1 to 5.8	0.860

^1^ LDH—lactate dehydrogenase; ^2^ N-MYC gene amplification; ^3^ INNS—International Neuroblastoma Staging System; ^4^ pOS—probability of overall survival; ^5^ HR—hazard ratio; ^6^ NA—not applicable; ^7^ CI—confidence interval; ^8^ pEFS—probability of event-free survival; ^9^ pRFS—probability of relapse-free survival. Structural chromosomal alterations were excluded from the multivariable analysis due to a low number of patients with aCGH test results; * *p* = 0.05–0.10.

## Data Availability

Data are available on reasonable request from the corresponding author.
